# Pitfalls in computer housekeeping by doctors and nurses in KwaZulu-Natal: No malicious intent

**DOI:** 10.1186/1472-6939-14-S1-S8

**Published:** 2013-12-19

**Authors:** Caron Jack, Yashik Singh, Maurice Mars

**Affiliations:** 1 Department of TeleHealth, Nelson R Mandela School of Medicine, University of KwaZulu-Natal, 719 Umbilo Road, Congella, Durban, South Africa

## Abstract

**Introduction:**

Information and communication technologies are becoming an integral part of medical practice, research and administration and their use will grow as telemedicine and electronic medical record use become part of routine practice. Security in maintaining patient data is important and there is a statuary obligation to do so, but few health professionals have been trained on how to achieve this. There is no information on the use of computers and email by doctors and nurses in South Africa in the workplace and at home, and whether their current computer practices meets legal and ethical requirements. The aims of this study were to determine the use of computers by healthcare practitioners in the workplace and home; the use and approach to data storage, encryption and security of patient data and patient email; and the use of informed consent to transmit data by email.

**Methods:**

A self-administered questionnaire was administered to 400 health care providers from the state and private health care sectors. The questionnaire covered computer use in the workplace and at home, sharing of computers, data encryption and storage, email use, encryption of emails and storage, and the use of informed consent for email communication.

**Results:**

193 doctors and 207 nurses in the private and public sectors completed the questionnaire. Forty (10%) of participants do not use a computer. A third of health professionals were the only users of computers at work or at home. One hundred and ninety-eight respondents (55%) did not know if the data on the computers were encrypted, 132 (36.7%) knew that the data were not encrypted and 30 (8.3%) individuals knew that the data on the computers they were using were encrypted. Few doctors, 58 (16%), received emails from patients, with doctors more likely to receive emails from patients than nurses (p = 0.0025). Thirty-one percent of individuals did not respond to the emails. Emails were saved by 40 (69%) recipients but only 5 (12.5%) doctors encrypted the messages, 19 (47.5%) individuals knowingly did not encrypt and 16 (40.0%) did not know if they encrypted the data. While 20% of health professionals have emailed patient data, but only 41.7% gained consent to do so.

**Conclusions:**

Most health professionals as sampled in South Africa are not compliant with the National Health Act or the Electronic Communications Transactions Act of South Africa or guidelines from regulatory bodies when managing patient data on computers. Many appear ignorant or lack the ability to comply with simple data security procedures.

## Introduction

Information and communication technologies (ICT) are changing the way we talk, think and communicate. ICT has become an integral part of medical practice, research and administration. Computers, cellular phones, tablet devices, short message service systems and fax machines are used to send patient related information between practitioners, patients and practitioners, and to hospital information systems and insurers [1,2]. These electronic communications form part of the patient's record. As such there is a statutory requirement to store the communications in a secure manner [3].

While doctors and nurses are aware of the need for secure storage of patient records to maintain confidentiality, the rapid increase in ICT use in the health sector poses potential threats of "data leakage" and unauthorised access to patient information [4]. There are many examples of sensitive information being hacked by cyber intruders or damaged by viruses [5]. More commonly in the health sector, data are lost through theft of notebook computers, tablets, flash drives or cellular phones [6]. The risk of data access following theft can largely be mitigated by encryption of stored patient data. Unauthorised access of emails may occur *en route*, between sender and recipient or when in storage on an email server or on the local machine. Patient information in databases may be compromised when passwords are shared between users or users fail to log off on completion of a task on a shared computer. Regulators and the public are naturally wary about potential breaches of privacy and confidential information falling into the wrong hands.

To minimise the risk and to ensure compliance with legislation and international guidelines on best practice of data management and security, sound basic computer housekeeping is required of health professionals. But few have had formal training to do so.

Although all medical schools in South Africa require students to use computers in their studies, little is known about computer use by doctors and nurses in South Africa. Masters (2008) found that 89% of general practitioners use computers, in a sample of 259 doctors [7]. Asah and others have reported low access to and use of computers by nurses in the public sector [8]. Nothing has been documented on encryption and storage by health professionals in South Africa.

The aims of this study were to determine the use of computers by healthcare practitioners in the workplace and home; the use and approach to data storage, encryption and security of patient data and patient email and the use of informed consent to transmit data by email.

## Methods

A questionnaire covering three domains was developed, addressing: demographic data; email use, security of emails in transit and storage, and the use of written informed consent for data transmission by email, and computer use by health professionals in the workplace and at home, sharing of computers, encryption and backup of data. The questionnaire was initially administered to several doctors and nurses for validation and to address any possible ambiguities, and then distributed to doctors and nurses for self-administration. Nurses were recruited at public and private hospitals and occupational health clinics and doctors were recruited at continuing professional development meetings. A sample size of 400 was chosen as per recommended guidelines for a knowledge practice attitude study and convenience sampling was used [9]. The study was undertaken with the approval of the Biomedical Research Ethics Committee of the University of KwaZulu-Natal and informed consent to participate was obtained from all subjects. No personal information was collected from participants therefore written informed consent was not a requirement

### Statistical analysis

All data were entered in an Excel spreadsheet and SPSS statistical package version 21 was used to perform the analysis. Descriptive statistics were used to describe the sample demographics. Fisher's exact test was used to compare results between doctors and nurses and the Chi square test for comparison between the three groups of doctors (specialists, general practitioners and medical officers). Alpha was set at 5%.

## Results

A total of 400 healthcare professionals completed the questionnaire, 193 doctors and 207 nurses. Of the doctors, 113 (58.5%) were male while 194 (93.7%) of nurses were female. The groups were further broken down into those who worked in public or private sector or both. The doctors were further divided into specialists, general practitioners (in private practice) and medical officers, the designation of general practitioners working in public sector hospitals. (Table 1)

**Table 1:** Demographic data of doctors and nurses by gender, clinical practice and work sector.

		Doctors n = 193(%)	Nurses n = 207(%)
**Gender**	Male	113(58.5)	13(6.2)

	Female	80(41.5)	194(93.7)

**Speciality**	Specialist	38(9.5)	

	General Practitioner	54(13.5)	

	Medical Officer	101(25.3)	

**Workplace**	Public Sector	111(57.5)	83(40)

	Private Sector	36(18.6)	99(47.8)

	Both	46(23.8)	25(12)

There were 40 (10%) people who do not use a computer in the workplace, practice or at home, 11 (5.7%) doctors and 29 (14%, p = 0.007) nurses. The non-users were excluded from further analysis relating to computer use. The responses for computer use, data backup and storage, and encryption are shown in Table 2 and the responses to questions on email communication with or by patients, storage and encryption of email and consent to send patient information electronically are shown in Table 3. A third of health professionals were the only users of computers at work or at home. One hundred and ninety-eight (55%) did not know if the data on the computers they use were encrypted, 132 (36.7%) knew that the data were not encrypted. Only 30 (8.3%) individuals knew that the data on the computers they use were encrypted.

**Table 2:** The positive responses of doctors and nurses to questions on their computer use, in the workplace and home and use of encryption and data backup.

	Nurses n = 178	Doctors n = 182	p	GP n = 51	MO n = 93	Specialist n = 38	p
**Use a computer in**	n (%)	n (%)		n (%)	n (%)	n (%)	

**Hospitals**	151 (84.8)	139 (76.4)	NS	29 (56.9)	75 (80.6)	35 (92.1)	0.0002

**Your Practice**	70 (39.3)	85 (46.7)	NS	42 (82.4)	22 (23.7)	21 (55.3)	<0.0001

**Home**	135 (75.8)	162 (89.0)	0.0016	42 (82.4)	84 (90.3)	36 (94.7)	NS

**If yes, only user?**							

**Hospitals**	40 (26.5)	15 (10.8)	0.0009	3 (10.3)	5 (6.7)	7 (20.0)	NS

**Practice**	33 (47.1)	26 (30.6)	0.049	8 (19.0)	5 (22.7)	13 (1.91)	0.0015

**Home**	39 (28.9)	55 (34.0)	NS	14 (33.3)	29 (34.5)	12 (33.3)	NS

**Data encrypted**	14 (7.9)	16 (8.8)	NS	1 (2.0)	6 (6.5)	4 (10.5)	NS

**Backup data**	48 (27.0)	69 (37.9)	0.0324	24 (47.1)	24 (25.8)	21 (55.3)	0.002

(GP-General Practitioner, MO-Medical Officer)

**Table 3:** The positive responses of doctors and nurses to questions on email use, storage, encryption and the use of consent, (%).(GP-General Practitioner, MO-Medical Officer)

	Nurses n = 178	Doctors n = 182	p	GP n = 51	MO n = 93	Specialist n = 38	p
**Do you receive emails from patients?**	18 (10.1)	40 (22.0)	0.0025	15 (29.4)	11 (11.8)	14 (36.8)	0.0023

**If yes do you answer them by email?**	11 (61.1)	29 (72.5)	NS	7 (46.7)	10 (90.9)	12 (85.7)	0.0173

**Do you store the emails?**	14 (77.7)	26 (65.0)	NS	11 (73.3)	7 (63.6)	8 (57.1)	NS

**If yes are they encrypted?**	1 (25.0)	4 (15.4)	NS	1 (9.1)	2 (28.6)	1 (12.5)	NS

**Have you emailed patient information?**	34 (19.1)	38 (20.9)	NS	6 (11.8)	14 (15.1)	18 (47.4	0.0001

**If yes do you get consent to do so?**	14 (41.2)	16 (42.1)	NS	3 (50.0)	5 (35.7)	8 (44.4)	NS

Very few healthcare providers, 58 (16%), received emails from patients, with doctors more likely to receive emails from patients than nurses (p = 0.0025), and specialists more likely to do so than their colleagues (p = 0.0023). Thirty-one percent of individuals/health professionals did not respond to these emails, with general practitioners least likely to reply (p = 0.0173). Emails were saved by 40 (69%) of recipients but only 5 (12.5%) individuals encrypted these. Nineteen (47.5%) individuals knowingly did not encrypt and 16 (40.0%) did not know if they encrypted the emails. Twenty percent of health professionals have emailed patient data to others, but less than half of them gained consent to do so.

## Discussion

The key findings of this study are that 90% of doctors and nurses use computers, either at home or in their workplace and approximately two thirds share computers with others. Although computer usage is high, few encrypt patient data saved on the computers or save the data to backup devices. Few individuals receive emails from patients but many store these without encrypting. Patient information therefore becomes vulnerable and at risk. It is unlikely that failure to encrypt transmitted and stored data is intentional. The health professionals in our study do not appear to know about encryption or its importance, as they are not doing it as routine practice.

In 2008, the Health Professions Council of South Africa which is the statutory body that governs medical practitioners published guidance on good clinical practice pertaining to confidentiality and keeping of patient records [10]. Likewise the South African Nursing Council has a code of ethical conduct, which refers to confidentiality. The HPCSA guidelines on keeping patients' records, which have to be kept for six years after the last consultation, merely state that the storage of records on CD-ROM is permissible provided that protective measures are in place, and that the records are encrypted and protected by passwords [10]. What they do not address is storage of patient information on home and workplace computers, computers with multiple users, differing levels of access rights to stored patient information by members of the health team, and guidelines on email communications and their secure storage. Studies have shown that patients and health care workers alike express concerns over privacy of health information when stored electronically and that they attach significant risk to these concerns [11,12].

The risk to data in patient information systems can be reduced by having at least four levels of security: 1) encryption or a similar technology for protecting confidentiality; 2) digital signatures and passwords or similar technology to ensure integrity, authentication and authorization; 3) a means to perform regular backups; and 4) disassociation of patient identifiers from patient data in a database.

The Canadian Medical Association has published guidelines regarding the use of email for healthcare communication, and cite three main areas of concern; confidentiality, privacy and security. The guidelines set out precautionary measures that need to be adhered to when communicating patient information via email and recommend obtaining written informed consent from patients prior to any email communication [13]. The Medical Protection Society of South Africa provides guidelines for doctors when communicating via email with patients and these guidelines also recommend obtaining written informed consent [14]. In this study, 20% of healthcare professionals email patient information to a third party, of whom 42% obtained written informed consent prior to doing so.

In South Africa an individual's right to privacy is enshrined in the South African Constitution. Section 14(4) of the Constitution states: "Everyone has the right to privacy, which includes the right not to have...the privacy of their communications infringed." In the health context, the patient's common law right to confidentiality has been codified and is explicitly recognized in section 14 of the National Health Act, 61 of 2003 [3].

The Electronic Communications and Transactions Act (ECTA), the first law governing cyber activity in South Africa, was promulgated in 2002. Broadly, this act provides for the facilitation and regulation of electronic communications and transactions. An electronic health record, an email correspondence containing patient information and a video-conferenced teleconsultation all meet the definition of an electronic transaction or communication [15]. Such data are termed "critical data" and are declared, in terms of section 53 to be, "... of importance to the protection of ....the economic and social well-being of its citizens."

Chapter 8 of the ECTA addresses the protection of personal information and sets out principles that must be adhered to when collecting such information. As these are voluntary principles, organisations do not have to adhere to them. The ECTA definition of personal information includes the following, "Information about an identifiable individual, including but not limited to- information relating to race, gender, and pregnancy," all of which can be deemed part of health related information. Clearly, most subjects in this study are not complying with the HPCSA guidelines or the ECTA. That they are wilfully transgressing is unlikely.

In 2011, the South African Department of Health released a white paper on the proposed national health insurance (NHI)[16]. The NHI aims to facilitate equitable medical services and standard of care to all long term residents of South Africa regardless of their financial status. The white paper refers to the use of centralised electronic patient health information systems by all health care professionals. This will require a still to be developed, national electronic medical record. The recently published eHealth strategy for South Africa 2012-2016 reconfirms South Africa's commitment to the use of all forms of information communication technologies to promote, support, and strengthen healthcare [17]. The use of hospital information systems in the public sector in South Africa is not new. A 2008 survey of electronic medical record systems in use showed that just over a third of the provincial hospitals have computerized systems in place but few of these are interoperable [18]. The Inkhosi Albert Luthuli Central Hospital in Durban, KwaZulu-Natal, is one of the few paperless hospitals in the World. Widespread use of ICT to manage patient information is inevitable and will involve all healthcare professionals, in both the public and private sectors.

In the private healthcare sector, a medical insurance company recently launched an application that allows doctors to electronically connect to the insurer's databases to access their patients' medical history, medical aid plans, laboratory results, write electronic prescriptions and make referrals to other healthcare professionals,. The insurer sees the use of the application in reducing diagnostic time, limiting medical error and reducing costs [19].

The move towards greater use of ICT in healthcare is in keeping with international trends where countries such as Australia and in the European Union are proposing centralized electronic health records and national databases that will allow inter jurisdictional access to medical records by healthcare professionals, insurers and governmental agencies in the country and across borders [20,21,23]. The HITECH Act in the United States and the EU's eHealth strategy will hasten widespread use of electronic medical records [22,24].

Various studies have investigated the threat of unauthorised access to patient data, citing lack of technical expertise and responsibility of health professionals [11,25]. To address these will require an appreciation of the risks to which medical information may be exposed, the development of robust policies for security, and raising the awareness of health professionals on these issues and providing further training for compliancy. Currently many are at risk, albeit unwittingly, of potential litigation.

## Conclusion

This study on the habits and practices in computer housekeeping of 400 healthcare workers in KwaZulu-Natal, has revealed areas of concern, these habits place patient information at risk of breaches of confidentiality. Knowledge or awareness around measures to ensure security of patient data is also lacking. A possible long term solution to this problem is to introduce basic medical informatics and telemedicine components in undergraduate medical school curricula. In the short term, seminars on managing electronic medical information and medical data security can be developed and should be part of continuing professional development assessment

## Abbreviations

ECTA: Electronic Communications and Transactions ACT; HITECH: Health Information Technology for Economic and Clinical Health Act; HPCSA: Health Professions Council of South Africa; SMS: Short Message Service.

## Competing interests

The authors declare that they have no competing interests.

## Authors' contributions

CJ conducted, designed the study, performed the primary analysis, collected the data and drafted the manuscript. MM conceptualized the study, interpreted the data, assisted in statistical analysis and revised the manuscript. YS assisted in statistical analysis and reviewed the manuscript.

## Declarations

Publication of this supplement has been funded by the College of Health Sciences and the Research Office at the University of Kwazulu-Natal.

This article has been published as part of *BMC Medical Ethics *Volume 14 Supplement 1, 2013: Selected papers from the 3rd Ethics, Human Rights and Medical Law Conference (3rd EHMRL). The full contents of the supplement are available online at http://www.biomedcentral.com/bmcmedethics/supplements/14/S1.
